# Advances in Digital Restorative Dentistry: Evaluation of Clinical Outcome Parameters of CAD/CAM and 3D‐Printed Inlays, Onlays, and Veneers—Scoping Review

**DOI:** 10.1155/ijod/7117281

**Published:** 2026-04-29

**Authors:** Syed Nahid Basheer, Syed Wali Peeran

**Affiliations:** ^1^ Department of Restorative Dental Sciences, College of Dentistry, Jazan University, Jazan, Saudi Arabia, jazanu.edu.sa; ^2^ Department of Preventive Dental Sciences, College of Dentistry, Jazan University, Jazan, Saudi Arabia, jazanu.edu.sa

**Keywords:** 3D printing, CAD/CAM, clinical outcome, digital dentistry, inlays, onlays, veneers

## Abstract

**Background:**

Digital workflows have revolutionized restorative dentistry, computer‐aided design and computer‐aided manufacturing (CAD/CAM) milling, and three‐dimensional (3D) printing provide alternatives to conventional methods of fabrication of inlays, onlays, and veneers. However, comparative evidence of their clinical performance remains scattered.

**Aim:**

This scoping review aimed to map and synthesize current evidence on the clinical outcomes of CAD/CAM– and 3D‐printed inlays, onlays, and veneers, focusing on adaptation, strength, esthetics, and workflow efficiency.

**Methodology:**

This scoping review followed the methodological framework proposed by Arksey and O’Malley, with refinements suggested by Levac et al., and was reported in accordance with the PRISMA‐ScR (Preferred Reporting Items for Systematic Reviews and Meta‐Analyses extension for Scoping Reviews). A comprehensive search was performed in PubMed, Scopus, Web of Science, Cochrane Library, and Google Scholar for studies published from January 2000 to June 2025. Eligible studies included in vitro, clinical, and case‐based reports that evaluated CAD/CAM or 3D‐printed inlays, onlays, or veneers. Data were charted and analyzed thematically according to restoration type, fabrication method, material, and reported outcomes.

**Result:**

Thirty studies were included, mainly in vitro. Inlays showed clinically acceptable adaptation, with 3D printing achieving accuracy comparable to milling, while ceramics offered superior hardness. Onlays yielded mixed outcomes: pressable ceramics had higher strength, but 3D‐printed onlays often showed better adaptation. Veneers demonstrated the greatest potential for 3D printing, with printed zirconia and lithium disilicate performing well. CAD/CAM was faster and well established, whereas 3D printing provided greater customization but longer fabrication times.

**Conclusion:**

In conclusion, both CAD/CAM and 3D printing are capable of producing inlays, onlays, and veneers with clinically acceptable accuracy and strength. CAD/CAM remains the most established and reliable technique, particularly for ceramics, whereas 3D printing shows potential to enhance adaptation and customizability, especially for veneers. Further clinical trials are essential to validate long‐term outcomes.

## 1. Introduction

Restorative dentistry has undergone a radical change in its landscape over the last couple of decades due to the influence of integrated digital technology and sophisticated manufacturing methods [1]. The most significant revolution among these new technologies has been the use of computer‐aided design and computer‐aided manufacturing (CAD/CAM) systems and three‐dimensional (3D) printing for transforming how clinicians manage indirect restorations like inlays, onlays, and veneers [2]. These protocols have been developed to enhance precision and efficiency in the fabrication process while allowing for material optimization and customization. However, current evidence remains inconclusive regarding whether 3D printing provides superior accuracy or efficiency compared to conventional CAD/CAM milling systems, particularly when postprocessing steps are considered. When compared to conventional workflows using laboratory‐fabricated restorations with multiple appointments, digital protocols enable clinicians to more precisely design and fabricate restorations with less chairside time and, in many cases, a single visit [3]. As these technologies evolve further, comparison of clinical performance of these technologies to conventional protocols is important and essential to yield evidence‐based decision‐making and optimize patient care.

Traditionally, inlays, onlays, and veneers have been produced from ceramics, composite resins, or metals with conventional impression‐taking, wax modeling, and lost‐wax casting techniques [4]. Although decades‐old technology has stood the test of times, it is restricted by operator variability, possible inaccuracies of impression‐taking, and tedious laboratory processing. The advent of CAD/CAM technology created a technology shift by allowing clinicians to digitally scan teeth they have prepared, virtually design restorations, and produce them with excellent precision by subtractive milling of ceramic or hybrid blocks [5]. With this system, human errors are minimized, the results are more reproducible, and the patients get the benefits of efficiency in the way they are treated. The more recent advent of 3D printing technologies opened the possibilities even wider. Rather than subtractive manufacturing, 3D printing involves additive manufacturing with the build‐up of restorations in thin layers using resins or ceramic‐molding materials, thus allowing complex geometries, negligible material waste, and potentially reduced production costs [6].

While milled restorations are supported by over three decades of clinical evidence, additively manufactured (3D‐printed) restorations remain an emerging area with limited long‐term clinical data. The marginal adaptation, fracture resistance, wear characteristics, esthetics, and long‐term survivability remain key determinants in the success of inlays, onlays, and veneers produced using these evolving digital technologies [7]. Additionally, there continue to be concerns about the biocompatibility and mechanical durability of newer printable resins, their staining resistance, as well as the effect of postprocessing operations on material characteristics [8]. In contrast, milled ceramics have a longer tradition of use and stronger clinical evidence, but they are flawed in terms of block size, inaccuracies in milling, and material waste [9]. Therefore, there is a need for systematic and comprehensive evaluation of contemporary evidence in order to grasp the extent to which the technologies meet their clinical promise.

Patient‐centered outcomes also merit special attention. For most patients, restorative care encompasses not only functional rehabilitation but also optimal esthetics, comfort, and convenience. Digital workflows—particularly chairside CAD/CAM milling units—offer significantly shorter treatment times, enabling same‐day restorations [10]. Such efficiency can enhance patient satisfaction by reducing the number of anesthetic administrations and minimizing the inconvenience of multiple appointments. Currently, chairside milled restorations demonstrate greater potential for esthetic personalization, as their surfaces can be customized through staining and glazing to achieve highly natural shade matching and translucency. In contrast, 3D printing technologies, while promising for rapid fabrication and material efficiency, are still limited in esthetic versatility, as integrated multichromatic shading within the printed material has not yet been fully developed. Nevertheless, ongoing research in multimaterial and color‐gradient printing is advancing toward overcoming these limitations [11]. Therefore, while 3D printing shows potential for efficiency and customization, its esthetic and clinical durability advantages over milled restorations remain areas requiring systematic investigation.

In recent years, several clinical investigations and systematic reviews have evaluated the performance of CAD/CAM–milled inlays, onlays, and veneers with satisfactory survival rates and acceptable complication profiles. The publications concerning 3D‐printed indirect restorations are continually available, but so far relatively few in number when compared with the evidence on CAD/CAM–milled inlays, onlays, and veneers, with the vast majority of publications being founded upon in‐laboratory mechanical and esthetic properties. This inconsistency highlights the importance of conducting a scoping review that charts the current evidence, defines the areas of research gap, and defines the current status of clinical effectiveness of CAD/CAM and 3D‐printed restorations. Through the integration of evidence from a large number of studies, such a review would offer significant insights into the relative effectiveness of such technologies, draw attention to the areas that demand more research, and inform both practice at the clinician level and future research directions. This scoping review aimed to map and synthesize current evidence on the clinical outcomes of CAD/CAM– and 3D‐printed inlays, onlays, and veneers, focusing on adaptation, strength, esthetics, and workflow efficiency.

## 2. Materials and Methods

This scoping review followed the methodological framework proposed by Arksey and O’Malley [12], with refinements suggested by Levac et al. [13], and was reported in accordance with the PRISMA‐ScR (Preferred Reporting Items for Systematic Reviews and Meta‐Analyses extension for Scoping Reviews) checklist.

### 2.1. Research Question and Protocol

This scoping review was conducted to systematically map and evaluate the clinical performance of inlays, onlays, and veneers fabricated using CAD/CAM and 3D printing technologies. The guiding research question was: “What is the current evidence on the clinical outcomes and performance of CAD/CAM and 3D‐printed inlays, onlays, and veneers in restorative dentistry?”

### 2.2. Eligibility Criteria

#### 2.2.1. Inclusion Criteria

• Studies were included if they focused on the development, performance, or clinical applicability of CAD/CAM or 3D‐printed inlays, onlays, and veneers in either human subjects or in vitro experimental models simulating clinical conditions.
• Clinical investigations (randomized controlled trials, cohort, case–control, and prospective or retrospective observational case series studies).
• Reports of clinical end points like survival rate, marginal adaptation, fracture resistance, esthetics, patient satisfaction, or complication rate.
• Original articles in English from refereed publications.
• Investigations in human subjects of any age or sex.

#### 2.2.2. Exclusion Criteria

• Technical reports, narrative review, systematic review, meta‐analysis, letter, editorial, or abstract at a meeting.
• Studies not specifically on CAD/CAM– or 3D‐printed inlays, onlays, or veneers.
• Non‐English language publications.

### 2.3. Information Sources and Search Strategy

A comprehensive search was conducted following the Arksey and O’Malley [12] framework and PRISMA‐ScR guidelines across PubMed, Scopus, Web of Science, and the Cochrane Library, with Google Scholar searched for gray literature (theses, preprints, and early‐access articles)—are summarized in Table 1. The search combined MeSH terms and free‐text keywords related to digital dentistry, CAD/CAM, 3D printing, additive manufacturing, and indirect restorations. Boolean operators (AND, OR) and truncations were applied to include variations in terminology.

**Table 1:** Searched strategies and source information.

Database	Search strategies	Results
PubMed	(“CAD/CAM”[MeSH] OR “Computer‐Aided Design” OR “Computer‐Aided Manufacturing” OR “Digital Dentistry” OR “3D Printing” OR “Additive Manufacturing”) AND (“Inlays” OR “Onlays” OR “Veneers”) AND (“Clinical Performance” OR “Longevity” OR “Survival Rate” OR “Marginal Adaptation” OR “Fracture Resistance” OR “Esthetics”)	345
Scopus	TITLE‐ABS ((“CAD/CAM” OR “3D Printing” OR “Additive Manufacturing” OR “Digital Workflow”) AND (“Inlays” OR “Onlays” OR “Veneers”) AND (“Clinical Outcomes” OR “Survival Rate” OR “Fracture Strength” OR “Patient Satisfaction”))	512
Web of Science	TS = ((“CAD/CAM” OR “Computer Aided Design” OR “Computer Aided Manufacturing” OR “3D Printing” OR “Additive Manufacturing”) AND (“Inlays” OR “Onlays” OR “Veneers”) AND (“Clinical Performance” OR “Survival” OR “Esthetics” OR “Marginal Integrity”))	274
Cochrane Library	(“CAD/CAM” OR “3D Printing” OR “Digital Dentistry”) AND (“Inlays” OR “Onlays” OR “Veneers”) AND (“Clinical Trials” OR “Survival” OR “Restorative Outcomes”)	185
Google Scholar	(“CAD/CAM Dentistry” OR “3D Printing in Dentistry” OR “Additive Manufacturing”) AND (“Indirect Restorations” OR “Inlays” OR “Onlays” OR “Veneers”) AND (“Clinical Outcomes” OR “Survival Rate” OR “Esthetic Dentistry”)	250
Total	—	1566

Key terms included: “CAD/CAM,” “Computer‐Aided Design,” “Computer‐Aided Manufacturing,” “Digital Dentistry,” “3D Printing,” “Additive Manufacturing,” “Inlays,” “Onlays,” “Veneers,” “Indirect Restorations,” “Lithium Disilicate,” “Zirconia,” “Hybrid Ceramics,” “PEEK,” “Fracture Resistance,” “Marginal Adaptation,” “Esthetics,” “Clinical Performance,” and “Longevity.”

The search was limited to English‐language studies (2000–2025) evaluating CAD/CAM or 3D‐printed inlays, onlays, and veneers in clinical or laboratory settings. Reviews, editorials, conference abstracts, and unrelated studies were excluded. This comprehensive approach ensured methodological transparency and inclusion of all relevant contemporary evidence.

### 2.4. Selection of Sources of Evidence

All retrieved records were exported into a reference management software, and duplicates were removed. Abstracts and titles were independently screened by two reviewers (Syed Nahid Basheer and Syed Wali Peeran) using the eligibility criteria. The full‐text screening then occurred on potentially relevant studies. Further disagreements were addressed by discussion or by consulting a third reviewer. The selection process was reported in a PRISMA flow diagram with numbers of identified, screened, excluded, and included records.

### 2.5. Data Charting Process

A systematic and standardized data charting process was employed to ensure accuracy and consistency in extracting information from the included studies. Two independent reviewers conducted the data extraction process using a predesigned and pilot‐tested data charting sheet. The charting form included essential study attributes such as authors, year of publication, country, study design, sample size, type of restoration (inlay, onlay, and veneer), fabrication method (CAD/CAM milling or 3D printing), material used, evaluated outcomes (e.g., marginal adaptation, fracture resistance, esthetics, and workflow efficiency), and key findings.

The reviewers independently extracted data from each eligible study. After extraction, the results were compared to ensure consistency. Any discrepancies or disagreements between the two reviewers were resolved through discussion and consensus, and if needed, a third reviewer was consulted to provide an impartial decision. This multireviewer approach minimized the risk of bias and improved the reliability of the data synthesis. The finalized data are organized into Tables 2–4 to provide a structured overview of the included studies. The extracted information was then synthesized narratively, highlighting key patterns, differences in methodologies, and trends in clinical and laboratory outcomes between CAD/CAM and 3D‐printed indirect restorations.

**Table 2:** Characteristics of included studies on CAD/CAM and 3D‐printed inlays.

Author/year	Country	Sample/study design	Material/technology	Clinical outcomes reported	Key findings
Sener‐Yamaner et al. (2017) [14]	Turkey	In vitro; 80 molars, 4 material groups	Pressed e.max (HLD), milled e.max (CLD), Lava Ultimate (NC), Filtek P60 (RC); two cement types	Marginal gap, cement thickness, fracture resistance	Milled e.max demonstrated the best marginal fit. Lava Ultimate had the highest fracture resistance, similar to sound teeth. Cement type did not significantly affect outcomes.
Homsy et al. (2018) [15]	Lebanon/Switzerland	In vitro; 75 lithium disilicate inlays (5 groups, *n* = 15)	Wax patterns: conventional/manual, milled, and 3D‐printed; pressed lithium disilicate	Marginal gap (μm): best with digital scan + milled wax; internal vs. marginal gap comparison	Digital scan with milled wax produced the best fit. 3D‐printed wax performed comparably to manual methods. Internal gaps were consistently larger than marginal gaps.
Ahlholm et al. (2019) [16]	Finland	In vitro; 6 molars with inlay/onlay cavities	Milled nanoceramic vs. 3D‐printed composite (Multijet); digital impressions (CEREC)	Internal gap measurements	3D‐printed restorations had 40%–60% smaller internal gaps than milled ones. Findings suggest 3D printing can achieve superior or equal fit compared to milling.
Prechtel et al. (2020) [17]	Germany	In vitro; 112 molars, 7 groups (*n* = 16); with/without fatigue	3D‐printed PEEK (4 brands), milled PEEK (Juvora), direct composite (Tetric), sound teeth	Fracture load (N)	3D‐printed and milled PEEK showed similar fracture strength, both higher than direct composites. Fatigue had little effect. Sound teeth had the greatest resistance.
Kim et al. (2021) [18]	South Korea	In vitro; 10 scans each for Class II inlays on maxillary and mandibular molars	CEREC Primescan; 3Shape lab scanner	Trueness and precision values	Maxillary molars showed better trueness than mandibular. Deviations were most frequent at proximal box margins.
Buduru et al. (2022) [19]	Romania	Case report; single tooth restored with both methods	Lithium disilicate: e.max press (conventional) vs. CAD/CAM workflow (Trios 3 + e.max CAD)	Esthetics, function, treatment time	Both workflows produced acceptable results. CAD/CAM was faster and less error‐prone, while conventional pressing allowed greater customization.
Özden et al. (2025) [20]	Turkey	In vitro; 180 inlays (3 resins × 3 postcuring times)	3D‐printed permanent resins (Crowntec, Bego, Senertek); LCD printer; postcuring at 2000/4000/6000 flashes	Trueness and precision at different depths	Crowntec had best trueness. 4000 flashes yielded most consistent accuracy. Both material and postcuring time significantly influenced results.
Tran et al. (2023) [21]	Vietnam	In vitro; 30 3D‐printed molars with Class II inlays at 3 cavity depths (*n* = 10/group)	Intraoral scanner; 3D‐printed resin models	Trueness and precision at different depths	Shallower cavities produced the most accurate scans. Deviations occurred mainly at gingival walls.
Karaoğlanoğlu et al. (2023) [22]	Turkey	In vitro; 96 specimens; CAD/CAM vs. 3D‐printed resins, immersed in tea, coffee, water	CAD/CAM: Grandio Blocs, Cerasmart 270; 3D‐printed: Crowntec, Permanent Crown	Microhardness, surface roughness, color stability	CAD/CAM blocks had superior hardness and color stability. Surface roughness was similar across groups. 3D‐printed resins showed more staining.
Lim et al. (2023) ([23]	South Korea	In vitro; 52 Class II inlays (4 groups, *n* = 13)	Conventional resin (TS), milled hybrid (LU), milled zirconia (ZR), 3D‐printed resin (NextDent)	Marginal gap, internal gap, trueness, precision	3D‐printed and zirconia inlays showed the best fit and accuracy.

**Table 3:** Characteristics of included studies on CAD/CAM and 3D‐printed onlays.

Author/year	Country	Sample/study design	Material/technology	Clinical outcomes reported	Key findings
Yildiz et al. (2013) [44]	Turkey	In vitro; 50 maxillary first molars restored with onlays	IPS e.max Press vs. IPS e.max CAD; cemented with SyntacVariolink (etch‐and‐rinse) or Multilink Sprint (self‐adhesive)	Fracture resistance	Pressable ceramics had higher fracture resistance than CAD/CAM onlays (*p* < 0.05). Etch‐and‐rinse cement performed better than self‐adhesive cement. All results were within clinical acceptability.
Revilla‐León et al. (2018) [35]	USA	In vitro; 12 onlay patterns (3 groups: handmade, milled, additive manufactured)	Handmade wax, milled wax (CAD/CAM), 3D‐printed resin	Marginal and internal gaps (CT scan, 1440 measurements)	Handmade wax yielded the lowest marginal (67.56 ± 6.08 μm) and internal (80.62 ± 3.26 μm) gaps. Additive manufacturing showed better internal fit than milling. All groups were within acceptable limits (<100 μm).
Lima et al. (2018) [36]	Brazil	In vitro, onlay preparations scanned directly (plastic teeth) and indirectly (stone dies)	CAD/CAM nanoceramic resin blocks (Lava Ultimate) milled with CEREC 4.0 and MCX	Marginal adaptation at 18 locations (stereomicroscope, 25×)	Conventional prep with modified shoulder (1.2 mm) had better adaptation (59 ± 50 μm) than modified prep with flat cuspal reduction (69 ± 58 μm). Indirect digital scanning gave better buccal fit (42 ± 33 μm) than direct scanning (60 ± 39 μm). All values within clinical range.
Alenezi and Yehya (2021) [37]	—	In vitro; 10 typodont molars scanned with two scanners (ARCTICA AutoScan, CEREC Omnicam)	Additive: SLA and DLP; Subtractive: e.max CAD with KaVo Everest, inLab MC X5	Marginal fit (digital microscopy)	Marginal gaps ranged 59–84 μm. No significant difference between milling and 3D printing (*p* = 0.70 for SLA; *p* = 0.21 for DLP). All techniques achieved clinically acceptable accuracy.
Cantó‐Navés et al. (2023) [38]	Spain	In vitro; 44 onlays (22 milled, 22 printed)	Group 1: milled graphene‐reinforced PMMA; Group 2: 3D‐printed hybrid composite	Marginal, central, and intaglio gaps; reproducibility	3D‐printed onlays had significantly better fit and reproducibility compared to milled counterparts (*p* < 0.05). Both groups shared the same CAD design.
Toma et al. (2023) [39]	—	In vitro study; 24 MOD onlays on 3D‐printed resin teeth (*n* = 8 per group)	High‐performance polymers milled with CAD/CAM: PEKK (Pekkton ivory), unmodified PEEK (Juvora medical), modified PEEK (BioHPP)	Marginal gap (MG) and internal gap (IG) assessed using micro‐CT	All materials showed MG < IG. BioHPP had the best marginal and internal fit. Significant differences observed among materials (except Juvora vs. BioHPP). Highest IG found at axiogingival line angles. All materials were within clinically acceptable adaptation limits.
Pasha et al. (2023) [40]	India	In vitro; 20 extracted mandibular first molars	CAD/CAM vs. 3D‐printed onlays; intraoral scans with Shining 3D scanner	Internal adaptation, marginal fit (stereomicroscope, micro‐CT)	3D‐printed onlays showed higher accuracy (*p* < 0.001), but CAD/CAM had better internal adaptation and marginal fit (*p* < 0.001 and *p* < 0.005). Both workflows followed the same design.
Vichi et al. (2023) [41]	UK	In vitro; 150 specimens from 8 composites (7 CAD/CAM, 1 printable)	CAD/CAM: Tetric CAD, Shofu Block HC, Cerasmart, Brilliant Crios, Grandio Bloc, Lava Ultimate, Katana Avencia; printable: permanent crown resin	Optical properties: translucency (CR, TP, TP00)	Translucency varied significantly. Katana Avencia (OP) was least translucent; Cerasmart (HT) most translucent. Findings suggest clinical caution when choosing materials for esthetic needs.
Li et al. (2024) [42]	China	In vitro; 40 mandibular first premolars with two prep designs	Nanoceramic resin; digital vs. conventional impressions; shoulder vs. no‐shoulder	Marginal adaptation	Digital impressions showed smaller marginal gaps than conventional (*p* < 0.05). Shoulder prep improved adaptation regardless of impression type (*p* < 0.05).
Waz and Alim (2025) [43]	Egypt	In vitro; 40 onlays (20 per group)	Group 1: 3D‐printed composite resin (NextDent C&B); Group 2: hybrid ceramic (Vita Enamic)	Fracture resistance (universal testing machine)	Vita Enamiconlays had higher fracture resistance than 3D‐printed composite, though not statistically significant (*p* > 0.05). Both materials performed comparably for premolar onlays.

**Table 4:** Characteristics of included studies on CAD/CAM and 3D‐printed veneers.

Author/year	Country	Sample/study design	Material/technology	Clinical outcomes reported	Key findings
Ioannidis et al. (2020) [24]	Switzerland	60 extracted human molars, in vitro experimental study	(1) 3D‐printed zirconia (Lithoz), (2) CAD/CAM milled zirconia (CeramillZolid FX), (3) heat‐pressed lithium disilicate (IPS e.max Press)	Finitial (initial crack load) and Fmax (fracture load) after fatigue testing	3D‐printed zirconia showed the highest load‐bearing capacity (*F*_initial_: 1650 N; *F*_max_: 2026 N). All materials exceeded functional thresholds, confirming clinical feasibility of ultrathin occlusal veneers.
Ioannidis et al. (2022) [25]	Switzerland	60 extracted human molars; in vitro comparative study	3D‐printed zirconia, CAD/CAM milled zirconia, heat‐pressed lithium disilicate	Marginal fit, internal fit, trueness	CAD/CAM zirconia achieved the best overall fit. 3D‐printed zirconia demonstrated clinically acceptable marginal adaptation (~95 μm) and trueness (~26 μm). Significant differences were noted in internal fit across groups.
Guachetá et al. (2022) [26]	USA	In vitro study using a typodont central incisor model	Pressed lithium disilicate veneers using 3D‐printed wax vs. manual waxing	Marginal gap and internal gap measurements	No significant difference between 3D‐printed wax and manual wax methods (*p* > 0.05). Both workflows provided clinically acceptable fit, supporting digital techniques as viable alternatives.
Robles et al. (2023) [27]	Mexico	Case report (single female patient)	3D‐printed additive CAD/CAM reduction guide with vertical/horizontal channels	Tooth preparation efficiency, esthetics, tooth preservation	The 3D‐printed guide enabled conservative preparation and real‐time evaluation, improving access and flexibility compared to conventional silicone guides. Promising for minimally invasive veneer cases.
Anghel‐Lorinți et al. (2024) [28]	Romania	60 veneer preparations; in vitro experimental study	3D‐printed provisional veneers across three finish line types (butt joint, feather edge, palatal chamfer)	Trueness, internal fit	Butt joint finish lines achieved the best overall accuracy. Differences among finish line designs were minor, suggesting preparation design influences but does not drastically alter fit.
Paqué et al. (2024) [29]	Switzerland	80 extracted human molars; in vitro experimental study (*n* = 20/group)	Lithium disilicate veneers fabricated via CAM milling, heat‐pressed PMMA, 3D printing, and heat‐pressed from 3D‐printed template	Load‐bearing capacity, internal accuracy, fabrication time	No significant difference in load‐bearing capacity (1687–2003 N; *p* = 0.5902). CAD/CAM was most time‐efficient (67 min), while 3D printing required the longest (701 min). Internal accuracy was lower in 3D‐printed groups.
Metin et al. (2024) [30]	Germany	In vitro study: 50 restorations (crowns, table‐tops, veneers) printed at 5 build angles (0°, 30°, 45°, 60°, 90°)	DLP 3D printing (Varseo XS) with hybrid resin‐ceramic	Trueness and precision (RMSE)	Build angle strongly influenced accuracy. Optimal orientation for veneers was 30°. The poorest results occurred at 90°, highlighting the need for angle optimization in printing.
Kalman and Tribst (2024) [31]	Canada	Pilot in vitro study using two demonstration casts; comparisons of milled vs. 3D‐printed zirconia	Milled vs. 3D‐printed zirconia veneers and crowns (Exocad design; Straumann scanning)	Fit, reproducibility, clinical acceptability	No significant differences between milled and 3D‐printed zirconia. Additive manufacturing produced consistent outcomes within clinical standards, supporting its future integration.
Elkaffas et al. (2025) [32]	Saudi Arabia	60 specimens; in vitro experimental study	Milled resin‐based ceramic (Cerasmart) vs. 3D‐printed resin‐based ceramic (VarseoSmile Crown plus A3); Luted with RC, BF, or PN	Fracture resistance	Both material and cement type significantly influenced fracture resistance (*p* < 0.001). Best performance: milled ceramic + PN. Lowest: 3D‐printed + BF. Cement selection is critical for clinical strength.
Daghrery et al. (2025) [33]	Saudi Arabia	30 extracted maxillary central incisors; in vitro comparative study	3D‐printed veneers (VarseosmileTrinQ, Varseosmile Crown Plus) vs. CAD/CAM milled (Brilliant Crios)	Marginal discrepancy, internal gap, overall adaptation	3D‐printed veneers had lower discrepancies and superior adaptation compared to milled veneers (*p* = 0.001). Brilliant Crios showed the largest gaps. Printing demonstrated better clinical fit.

### 2.6. Synthesis of Results

The extracted data were compiled and summarized descriptively to provide an overall summary of current evidence. Results were then ranked thematically by restoration class, fabrication method, material, and clinician‐reported clinical outcome. As a heterogeneity of study design and outcome instrument variation, a formal meta‐analysis could not be performed.

### 2.7. Consultation With Expert

To enhance validity and clinical relevance of findings, an expert consultation stage was included within review planning. An expert with extensive experience with digital workflows in restorative dentistry was consulted to present his opinion about how to interpret results and to point out missing perspectives.

## 3. Results

### 3.1. Study Selection

**Figure 1:**
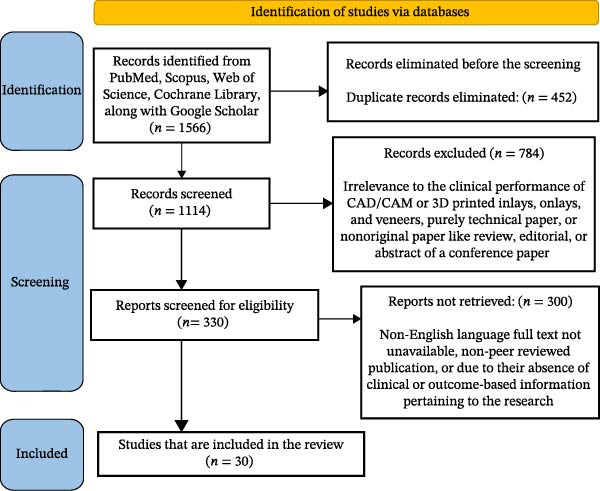
PRISMA flowchart demonstrates the screening process of studies retrieved from different web sources.

A total of 1566 records were initially identified through PubMed, Scopus, Web of Science, Cochrane Library, and Google Scholar. Thereafter, 452 duplicate records were excluded, and title and abstract screening were conducted on 1114 articles. Of these, 784 articles were excluded due to their irrelevance to the clinical performance of CAD/CAM or 3D‐printed inlays, onlays, and veneers, purely technical paper, or nonoriginal paper like review, editorial, or abstract of a conference paper. The remaining 330 papers were subject to full‐text evaluation according to eligibility. At this stage, 300 papers were excluded because they were not in English, full text not unavailable, non‐peer‐reviewed publication, or due to their absence of clinical or outcome‐based information pertaining to the research. Finally, 30 papers were included to undergo the final synthesis of this scoping review. The study identification, screening, and inclusion process are shown in the PRISMA flowchart (Figure 1).

### 3.2. Characteristics of Studies Included

The majority of studies were in vitro laboratory experiments (*n* = 27), with only a small number of case reports (*n* = 3). Studies were geographically varied and were from Europe, Asia, North America, and the Middle East, reflecting the international implementation of CAD/CAM and 3D‐printing technologies in restorative dentistry. Sample sizes in the included papers varied from single clinical case reports through to matched laboratory experiments with 180 specimens. Although inlays, onlays, and veneers were all represented, inlays were the most thoroughly investigated group, followed by onlays and veneers.

In terms of fabrication methods, the evidence compared traditional press and manual processes against CAD/CAM subtractive milling and 3D printing additive processes. Most common testing materials were lithium disilicate ceramics, resin‐based composites, zirconia, and PEEK/PEKK–based polymers, with more contemporary investigations also testing hybrid ceramic and printable resin‐based dentures and restorations. Presented clinical outcomes were marginal and internal adaptation, trueness and precision of digital processes, fracture resistance, microhardness, esthetics (translucency and color stability), surface roughness, and fabrication time. Collectively, the evidence provides an overview, but heterogeneous set of information concerning the digital technologies compared to traditional technologies.

Of the 30 studies included in this scoping review, 27 were in vitro laboratory investigations, three were case reports, and none were large‐scale clinical trials. The inclusion of in vitro studies was essential to capture fundamental data on material behavior, marginal adaptation, and mechanical performance under standardized conditions, which form the basis for clinical translation. Case reports, though limited in generalizability, were included because they provide valuable real‐world insights into the early clinical feasibility and patient‐centered applications of CAD/CAM and 3D‐printing technologies for indirect restorations. This mixed inclusion of study types offers a comprehensive overview of both laboratory evidence and preliminary clinical experience within the scope of emerging digital restorative workflows. Therefore, findings related to adaptation, strength, and esthetics should be interpreted cautiously when extrapolated to patient care.

### 3.3. Extent, Nature, and Distribution of the Evidence

Both CAD/CAM and 3D printing techniques produced restorations within clinically acceptable marginal and internal adaptation limits. In accordance with established literature and ISO guidelines [34], a marginal gap of up to 120 μm is generally considered clinically acceptable for indirect restorations, as it ensures proper sealing, minimal cement dissolution, and long‐term restoration success. Fracture resistance values varied according to material type, with milled lithium disilicate ceramics exhibiting the highest mean values (~1800–2500 N) and printed resin‐based or hybrid composites showing moderately lower values (900–1600 N). Fabrication times were shorter for CAD/CAM systems (45–90 min) compared with 3D printing workflows (90–150 min including postprocessing). These findings indicate that both methods can achieve clinically acceptable precision, though performance differences are material‐dependent.

The degree of evidence delivery varied significantly with restoration type.

#### 3.3.1. Inlays

These investigations primarily discussed fit (internal and marginal gaps), resistance to fracture, and scanning accuracy [14–23]. Characteristics of included studies on CAD/CAM and 3D‐printed inlays are summarized in Table 2. Several studies demonstrated 3D printing to achieve equal or superior precision with respect to milling [16, 21], while CAD/CAM–milled ceramics tended to achieve higher hardness and color stability [22, 23]. Clinical evidence was scarce, but suggested CAD/CAM had efficiency advantages, with conventional pressing techniques still achieving higher customization potential.

#### 3.3.2. Onlays

The evidence base highlighted fracture resistance and marginal adaptation as major outcomes [35–43]. Characteristics of included studies on CAD/CAM and 3D‐printed onlays are summarized in Table 3. Previous studies showed pressable ceramics were better compared to milled lithium disilicate, but subsequent comparisons showed 3D‐printed onlays more likely to achieve improved fit in comparison with milled counterparts [44]. More recent comparisons indicated that 3D‐printed onlays frequently achieved superior fit and reproducibility relative to milled alternatives, though results varied with material, printing method, and preparation design [35, 38–40]. Several investigations also highlighted the influence of cementation protocols and digital scanning modalities on adaptation quality [36, 37, 42].

#### 3.3.3. Veneers

Studies on veneers explored load‐bearing ability, marginal and internal fit, optical properties, and preparation accuracy [24–33]. Characteristics of included studies on CAD/CAM and 3D‐printed veneers are summarized in Table 4. Early research established the potential of ultrathin occlusal veneers across different materials [24], while recent research showed 3D‐printed veneers were possible with smaller discrepancies and improved general adaptation compared with milled counterparts [27, 28, 32, 33]. Studies evaluating optical and esthetic outcomes revealed considerable variability among CAD/CAM composites, reinforcing the importance of careful material selection [30, 41]. A small number of case reports illustrated the feasibility of additive techniques for clinical use, including digital reduction guides and provisional veneers [27, 32].

### 3.4. Thematic Analysis

The numerical data across studies underscore distinct trade‐offs between fabrication technologies. CAD/CAM milling consistently demonstrated higher fracture strength (1500–2500 N) and faster turnaround (under 90 min), confirming its reliability for high‐stress restorations. In contrast, 3D printing provided superior adaptability and surface detail, maintaining marginal fit values within 40–100 μm, but generally required longer fabrication times (up to 150 min) and showed reduced mechanical endurance (900–1800 N). This comparison highlights that while 3D printing enhances customization and precision, CAD/CAM remains superior for mechanical reliability and workflow efficiency.

Thematic synthesis revealed differences based on restoration type, fabrication method, material, and outcome measures. For inlays, CAD/CAM and 3D printing produced clinically compliant marginal gaps (<120 μm), with zirconia and 3D‐printed resin routinely outperforming hybrid composites in precision [14–23]. Greater variability was apparent with onlays: pressable ceramics produced greater fracture resistance compared with milled lithium disilicate [44], while more recent studies determined 3D‐printed onlays demonstrated better adaptation and reproduction compared with their milled counterparts, but with material‐dependent performance [35–43]. Veneers demonstrated promising results for 3D‐printed zirconia and lithium disilicate, which routinely matched or surpassed their milled counterparts in adaptation and strength [24–29]. Case reports also confirmed the clinical viability of 3D printing in conservative veneer preparation and temporary restorations [27, 32].

By fabrication method, CAD/CAM milling offered speed, high hardness, and color stability, but some studies reported larger internal misfits [16, 23, 43]. 3D printing permitted improved adaptability and customizability [21, 26, 38, 43], although lower hardness, staining resistance, and longer fabrication time [21, 39]. Material‐wise, lithium disilicate ceramics still represented the gold standard for esthetic and strength [19, 23, 44], zirconia (especially 3D‐printed) demonstrated excellent load‐bearing capacity [25, 26], and hybrid ceramics, composites, and PEEK/PEKK polymers demonstrated fair performance but lower durability [17, 22, 41].

Across clinical outcomes, fit and accuracy were most reported, and both techniques resulted in clinically acceptable values [15, 16, 35, 38, 40]. Ceramics always excelled over resin‐based systems in fracture resistance [25, 32, 41, 44], whereas esthetic results vacillated extensively with composites [21, 41]. Workflow convenience preferred CAD/CAM milling for quick chairside delivery, whereas 3D printing was beneficial for design versatility, but required longer processing times [29]. Overall, both methods create restorations within clinical parameters, yet high‐quality clinical trials are essential to validate long‐term success.

## 4. Discussion

The aim of this scoping review was to map existing evidence of the clinical performance of CAD/CAM and 3D‐printed inlays, onlays, and veneers. Thirty studies included most of which were in vitro, contributing a few case reports with limited clinical evidence. The findings confirm that digital workflows inevitably generate restorations within clinically acceptable limits, but clear advantages and limitations ended up being dependent on restoration type, fabrication technology, and material.

It is important to note that the included studies displayed substantial heterogeneity in terms of study design, materials, fabrication workflows, and evaluation parameters. The evidence spanned in vitro experiments, case reports, and limited clinical studies, each using varied materials such as ceramics, composites, and hybrid resins, as well as different performance indicators including marginal adaptation, fracture strength, and esthetic quality. This variability limited the possibility of direct comparison or quantitative synthesis. However, such heterogeneity reflects the rapidly evolving nature of digital restorative dentistry, where diverse materials and technologies continue to emerge. Future research should aim for greater standardization in study design, testing protocols, and outcome reporting to facilitate comparability and strengthen the evidence base.

### 4.1. Inlays

Inlays were the most widely investigated restorations, and findings have focused on marginal and internal adaptation, fracture resistance, and accuracy of digital workflows [14–23]. Several studies confirmed that both CAD/CAM and 3D printing produced restorations with marginal gaps smaller than the clinically acceptable threshold of ≤120 μm, as recommended by previous research for optimal clinical performance and longevity [14, 15, 18, 23, 34]. A few studies reported smaller internal gaps being realized for 3D‐printed inlays compared to milled ones [16, 21], a finding consistent with the advantages of the process of additive fabrication to reproduce complex geometries. Although there are favorable outcomes, milled ceramics such as lithium disilicate and zirconia consistently showed higher hardness, better color stability, and predictable esthetics than 3D‐printed resins [22, 23]. This finding agrees with earlier systematic reviews [45–47], referring to the mechanical superiority of ceramics to resin‐based CAD/CAM systems. In comparison, polymer alternatives such as PEEK and PEKK showed moderate fracture resistance and durability [17], providing broader restorative choices for specific indications. The limited clinical evidence, such as the case report of Buduru et al. [19], indicated that CAD/CAM inlay reduced treatment time and potential error, yet pressed lithium disilicate still offered greater opportunities for customization. These findings reflect the compromise between the digitized expediency and the expertise of the conventional pressing technique.

### 4.2. Onlays

The literature on onlays showed a wider range of outcome [35–44]. Earlier work had already confirmed that pressable ceramics possessed a higher fracture resistance than milled lithium disilicate [44], a finding also reinforced by Yoon et al. [48]. Subsequent work, however, claimed that 3D‐printed onlays tended to exhibit enhanced marginal adaptation and reproducibility over milled counterparts [35, 38–40], showcasing the potential of addition‐type workflows for demanding restorations. Design of preparation and scanning modality were shown to significantly influence results. Lima et al. [36] reported indirect digital scanning to have smaller marginal gaps compared to direct intraoral scanning, whereas Li et al. [42] showed a better adaptation, irrespective of impression method, with shoulder preparation. These results resonate for the continuation of the fundamental principles of tooth preparation even in this age of digital dentistry.

Cementation protocols also influenced performance. Yildiz et al. [44] discovered etch‐and‐rinse cements to improve fracture resistance compared to self‐adhesive systems, the finding agrees with Eltoukhy et al. [49]. Similarly, Alenezi and Yehya [37] and Toma et al. [39] also confirmed material selection and choice of workflow significantly impacted marginal and inner adaptation. Altogether, these works suggest 3D printing precision advantages, but long‐term clinical success ultimately rests on preparation, adhesion, and restoration design.

### 4.3. Veneers

Veneers were less studied but provided some of the strongest evidence for the disruptive potential of 3D printing [24–33]. Ioannidis et al. [24] demonstrated 3D‐printed zirconia veneers had load‐baring capacity beyond functional limits and performed similarly or preferably to milled or pressed lithium disilicate. Other investigations also demonstrated similarly printed veneers had lesser deviations and improved adaptation compared to CAD/CAM–milled equivalents [27, 28, 33]. Both optical and esthetic performance varied significantly. Vichi et al. [41] stressed wide differences in translucency of CAD/CAM composites, and stressed material dependence of esthetic outcomes. This finding is consistent with previous literature [50, 51] for the range of translucency and color stability of indirect restorative composites.

Clinical feasibility was also illustrated in case reports. Robles et al. [27] documented that a 3D‐printed reduction guiding device enabled minimally invasive veneer preparations with improved accuracy relative to standard silicone guides. Such applications are consistent with the long‐standing trend of going towards guided, conservative dentistry. Even so, as in inlays and onlays, veneers do not have long‐term clinical validation, highlighting again the discrepancy between laboratory results and daily practice.

### 4.4. CAD/CAM Milling Versus 3D Printing

CAD/CAM milling and 3D printing emerged as complementary technologies. Milling remained the gold standard for speed, predictability, and validated ceramic outcomes [14, 19, 23, 44]. It provides same‐day restorations and has the best history of long‐term clinical success [2, 52]. Its weaknesses, however, are waste of material, wear for tools, and geometrical limitations. By contrast, 3D printing allows extremely customized and complex‐geometry restorations to be produced with reduced waste [16, 21, 27, 35, 38]. Flexibility was generally better in printed specimens [27, 33, 35, 38], but constraints of hardness, shade stability, and long‐term durability of printed resins remained a continuous drawback [22, 32, 43]. Fabrication times were also generally longer than for milling workflows [29]. These findings agree with recent systematic reviews [45, 53] reporting 3D printing as extremely accurate but constrained by material properties.

The enhanced adaptation and customization observed with 3D printing can be attributed to the intrinsic nature of additive manufacturing, which constructs restorations layer by layer directly from digital models. Unlike subtractive milling, which removes material from prefabricated blocks and is limited by bur size, tool wear, and milling path constraints, 3D printing reproduces intricate geometries and fine internal details with minimal material stress. The voxel‐based digital slicing process allows for precise control over layer thickness and localized density, improving the accuracy of internal and marginal fits. Furthermore, design modifications can be made easily before fabrication, facilitating individualized contours, occlusal morphology, and esthetic parameters tailored to each patient. These characteristics explain the improved adaptability and personalization seen in 3D‐printed restorations, particularly in veneers and anatomically demanding restorations where esthetic precision and minimal invasiveness are prioritized.

It is important to note that considerable heterogeneity exists among the included studies in terms of outcome definitions and measurement methodologies. Marginal adaptation was assessed using varied techniques such as microcomputed tomography, silicone replica, or digital scanning, while mechanical strength was measured through different testing protocols, including fracture load and flexural strength assessments. Similarly, esthetic evaluation methods ranged from subjective visual analysis to objective spectrophotometric measurements. These methodological variations limit the direct comparability of results and highlight the need for standardized testing and reporting protocols to enable meaningful synthesis across studies.

### 4.5. Material Considerations

A clear hierarchy of materials was evident. Lithium disilicate ceramics remained the most reliable for esthetic and mechanical properties, with pressed versions being superior to milled equivalents for fracture resistance [44]. Zirconia, especially in a printed version, provided a high load‐bearing capacity and trueness [25, 26], pointing toward increasing use in thin restorations. Hybrid ceramics and printable composites provided workflow versatility and moderate fit [28, 35, 38, 41], but consistently lagged behind ceramics for hardness and optical stability [22, 41]. Polymers such as PEEK and PEKK provided compromise performance, suggesting a permanent function for temporary applications or for implant‐secured applications [17, 39]. These findings reinforce existing reviews [54–56] on restorative materials, in which focus is on the durability of ceramics as the strongest material choice.

A clear distinction exists among different 3D printing technologies and materials used in indirect restorations. Resin‐based 3D printing, typically utilizing photopolymer resins through SLA or DLP, offers excellent precision but limited mechanical strength and color stability [57]. In contrast, ceramic‐based printing methods such as zirconia or lithium disilicate produced via slurry‐based or powder‐bed techniques provide superior hardness, durability, and esthetics, though they require complex postprocessing [58]. Hybrid printable composites combine workflow flexibility with moderate strength and are primarily suited for provisional or minimally loaded restorations [59]. These material‐dependent differences are crucial for selecting appropriate 3D printing systems in clinical applications.

### 4.6. Clinical Implications

Both workflows are clinically capable of producing an acceptable restorative result. CAD/CAM should be the preferred option for its efficient and long‐term proven performance and should be reserved for single‐unit ceramic restorations. 3D printing should be reserved for situations where there is a high degree of customization, minimally esthetic preparations, or unusual geometries. Anterior esthetic cases are particularly demanding due to the great variation of translucency and color stability of the material. Clinicians must incorporate the 3D printing process cautiously and using it to help augment existing CAD/CAM procedures until stronger clinical evidence can be developed.

### 4.7. Limitations and Future Directions

This scoping review has several important limitations that must be acknowledged. First, the predominance of in vitro studies, which restricts the direct translation of results to clinical practice. Laboratory experiments often assess performance under ideal conditions, lacking variables such as saliva, occlusal stress, and patient factors that influence restoration longevity. Consequently, the evidence base remains preliminary, highlighting the need for more well‐designed clinical trials to validate these findings. Second, there is a notable lack of long‐term clinical trials comparing CAD/CAM and 3D‐printed restorations. Most available clinical evidence consists of short‐term case reports or pilot studies, making it difficult to assess long‐term survival rates, esthetic stability, and patient‐reported outcomes. Third, the rapid technological evolution in digital dentistry presents a unique challenge. Many earlier studies used outdated printers, materials, or software, which may not reflect current or future clinical performance. Consequently, heterogeneity in study designs, materials, and testing protocols further complicates direct comparisons and synthesis of results. Formal risk‐of‐bias assessment was not performed, as this step lies beyond the typical scope of a scoping review. Instead, we ensured a level of quality assurance by including only peer‐reviewed studies and by documenting study design, sample characteristics, and methodological transparency during data extraction. Future research should include formal risk ‐of‐bias assessment and focus on well‐designed and multicenter clinical trials with standardized protocols to validate laboratory findings under clinical conditions. Continuous material innovation is also needed to enhance the mechanical strength, esthetic properties, and long‐term stability of printable ceramics and hybrid composites. Finally, establishing standardized reporting guidelines and exploring hybrid workflows that integrate the precision of CAD/CAM with the design flexibility of 3D printing could provide a more robust foundation for clinical application and evidence‐based adoption in restorative dentistry.

## 5. Conclusion

In conclusion, both CAD/CAM and 3D printing are capable of producing inlays, onlays, and veneers with clinically acceptable accuracy and strength. CAD/CAM remains the most established and reliable technique, particularly for ceramics, whereas 3D printing shows potential to enhance adaptation and customizability, especially for veneers. Although both CAD/CAM and 3D printing technologies show promising laboratory outcomes, the limited clinical evidence available warrants cautious interpretation. The current findings primarily reflect in vitro results, and future research should prioritize robust clinical trials to confirm long‐term performance, patient satisfaction, and real‐world applicability of these digital restorative methods.

## Funding

No funding was received for this manuscript.

## Conflicts of Interest

The authors declare no conflicts of interest.

## Data Availability

Data sharing is not applicable to this article as no datasets were generated or analyzed during the current study.
